# Pediococcus pentosaceus Endocarditis in a Patient With Recent Transcatheter Aortic Valve Implantation and Liver Cirrhosis: A Case Report and Review of the Literature

**DOI:** 10.7759/cureus.57509

**Published:** 2024-04-03

**Authors:** Petros G Mantzios, Panagiota Spyropoulou, Sophia Hatzianastasiou, Dimitrios Efthymiou, Efthymios Filippopoulos, Christos Mamarelis, Charalampos Potsios, Konstantina Filioti, Constantinos A Letsas

**Affiliations:** 1 Internal Medicine, General Hospital of Eastern Achaia, Aigio, GRC; 2 Infectious Diseases, Onassis Cardiac Surgery Center, Athens, GRC

**Keywords:** positron emission tomography, cirrhosis, transcatheter aortic valve implantation, infective endocarditis, pediococcus pentosaceus

## Abstract

Transcatheter aortic valve implantation (TAVI) is increasingly being used in the management of severe aortic stenosis, mainly in older and/or medically compromised patients, due to its minimally invasive nature. As in any valve replacement procedure, endocarditis is a recognized complication, more so in TAVI patients, in whom comorbidities are highly prevalent. We report the case of a 70-year-old male with a history of liver cirrhosis and a recent TAVI, who presented with recurrent fever and sustained*Pediococcus pentosaceus* bacteremia. The diagnosis of endocarditis was delayed, as the microorganism was initially discarded as a contaminant, given that *Pediococci* are rarely described as human pathogens. However, in cirrhotic patients, microbiota may cause intermittent bacteremia and thereby affect prosthetic valves. Transthoracic echocardiography was not helpful in validating the diagnosis, as is often the case in TAVI patients. Transesophageal echocardiography was deemed perilous, due to esophageal varices complicating the underlying cirrhosis. Therefore, endocarditis diagnosis was based on sustained bacteremia and Duke’s criteria, including the presence of high fever, a predisposing cardiac lesion, splenic infarction, and the exclusion of an alternative diagnosis. Moreover, cirrhosis enhanced the side effects of treatment and led to the need for regimen changes and prolonged hospitalization. Given the precariousness of the situation, confirmation of treatment success by 2-deoxy-2-[fluorine-18]fluoro-D-glucose positron emission tomography-computed tomography (18F-FDG PET-CT) scan was sought. This is the first reported case of *Pediococcus* TAVI endocarditis in a cirrhotic patient, highlighting the unique challenges in the diagnosis and management of TAVI endocarditis in patients with co-existing conditions.

## Introduction

Infective endocarditis (IE) after transcatheter aortic valve implantation (TAVI) is a rare, yet significant complication of this revolutionary and increasingly popular technique. It can be classified as early (within 60 days post-TAVI), intermediate (2-12 months post-TAVI), or late (>12 months post-TAVI) [1]. In most of the largest published cohorts, ​​​Enterococcus spp. has been identified as the most common pathogen, followed by Staphylococcus aureus. As a clinical entity, its incidence is relatively low, ranging from 0.1 to 3% [1-3]. However, post-TAVI IE carries a poor prognosis, not only in comparison to native valve endocarditis but, also, in surgically replaced valve endocarditis, regarding both valvular dysfunction and patient mortality [1]. This is a result of notoriously troubled echocardiographic diagnosis (due to acoustic shadowing artifacts caused by increased metal quantity compared to surgically placed valves) and non-specific symptoms along with the clinical profile of the majority of TAVI candidates, namely elderly and high surgical risk patients with a multitude of comorbidities [1]. These comorbidities may affect the type of microorganisms involved, promoting the deviation from common IE pathogens, for which there is extensive clinical experience, and enhancing the infectious potential of opportunistic pathogens, many of which are not addressed in current guidelines [4-6].

*Pediococci* are Gram-positive, catalase-negative bacteria [7,8]. They are characterized as lactic acid bacteria (LAB), due to their ability to produce lactate as the final product of carbohydrate fermentation [7,9]. *Pediococcus pentosaceus* strains are, also, contained in commercially available probiotics [7]. While *Pediococci* are normally harmless, symbiotic bacteria that contribute to maintaining homeostasis of the gut microbiome, they may rarely emerge as opportunistic pathogens in immunocompromised hosts, with potentially lethal complications [10-12]. Immune dysfunction is a hallmark feature of cirrhosis; multiple pathways lead to its development [13]. Herein, we present a case of IE caused by *P. pentosaceus* in a cirrhotic patient with a recent TAVI, the first documented so far.

## Case presentation

A 70-year-old Caucasian male presented to the Emergency Department (ED) with reported chills and high-grade fever (39^ο^C). Blood cultures were obtained, and he was discharged home on oral ciprofloxacin 500mg BID for 10 days, with a working diagnosis of urinary tract infection due to the presence of white blood cells in urine. After completion of the 10-day antimicrobial course, the fever rebounded, bringing him to the ED for the second time. When a second blood culture obtained upon admission grew a Gram-positive, non-hemolytic coccus identified as *P. pentosaceus*, the laboratory informed us that the same pathogen was isolated from the patient's initial blood culture drawn 10 days previously but was then discarded as a contaminant. He reported persistent, low-grade fever during the last three months, for which he sought no medical consultation since it subsided with self-administered antipyretics.

The patient's past medical history was significant for liver cirrhosis secondary to primary biliary cirrhosis (PBC) treated with ursodeoxycholic acid for the past seven years. His condition was complicated by portal hypertension and esophageal varices, last ligated five years ago, as well as an episode of hepatic encephalopathy. He had undergone TAVI four months ago and his thoracic aortic aneurysm had last been measured at 5.4cm. Additional history included previous laparoscopic cholecystectomy, osteoporosis, dyslipidemia, and benign prostatic hyperplasia. He was a non-smoker and did not report excessive alcohol consumption. 

At presentation, apart from low-grade fever (37.9^o^C), his vital signs were normal. Significant findings upon physical examination were pitting lower limb edema and hepatosplenomegaly, without other signs of chronic liver disease. There was no evidence of poor oral hygiene and he had not been submitted to a dental procedure within the last five years. Physical examination was negative for heart murmurs. Blood tests revealed polymorphonuclear leukocytosis, anemia, mild thrombocytopenia, and moderately reduced serum albumin levels, as well as elevated inflammatory markers (C-reactive protein, CRP, erythrocyte sedimentation rate, ESR) and increased levels of high-sensitivity Troponin I (hsTnI). Proteinuria was present, along with mild microscopic hematuria without dysmorphic RBCs on urinalysis. Liver function tests, as well as coagulation parameters, were within normal limits. Mild indirect hyperbilirubinemia was attributed to Gilbert’s syndrome. Results of the initial blood tests are summarized in Table 1. 

**Table 1:** Patient blood tests on admission. Pathologic results are presented in bold.

Lab parameter	Value	Reference ranges
Hemoglobin (Hgb)	10.9 g/dL	14-18 (for males)
Hematocrit (Hct)	34.4 %	42-52 (for males)
White blood cells (WBC)	12.00 K/μL	4.5-10
Neutrophils	10.99 (91.6%) K/μL	1.8-7
Lymphocytes	0.35 (2.9%) K/μL	1.2-3.8
Platelets (PLT)	100 K/μL	140-450
Aspartate aminotransferase (AST)	45 ΙU/L	0-40
Alanine aminotransferase (ALT)	22 IU/L	<45
Αlkaline phosphatase (ALP)	144 IU/L	30-120
γ-Glutamyl transferase (γ-GT)	44 IU/L	10-49
Total bilirubin	3.2 mg/dL	< 1.2
Direct bilirubin	0.5 mg/dl	<0.3 mg/dl
Indirect bilirubin	2.7	
Albumin (ALB)	2.8 gr/dL	3.4-4.8
Creatinine (Cre)	0.7 mg/dL	0.7-1.4
Urea	39 mg/dL	10-50
Sodium (Na+)	133 mmol/L	136-145
Potassium (K+)	3.8 mmol/L	3.5-5.2
High-sensitivity troponin I (HsTnI)	99.8 pg/mL	<34.2 (for males)
C-reactive protein (CRP)	7.39 mg/dL	<0.7
Erythrocyte sedimentation rate (ESR)	55 mm/Hr	<30
International normalized ratio (INR)	1.21	0.8-1.1
Prothrombin time (PT)	12.1 sec	9.1-12.1

**Figure 1:**
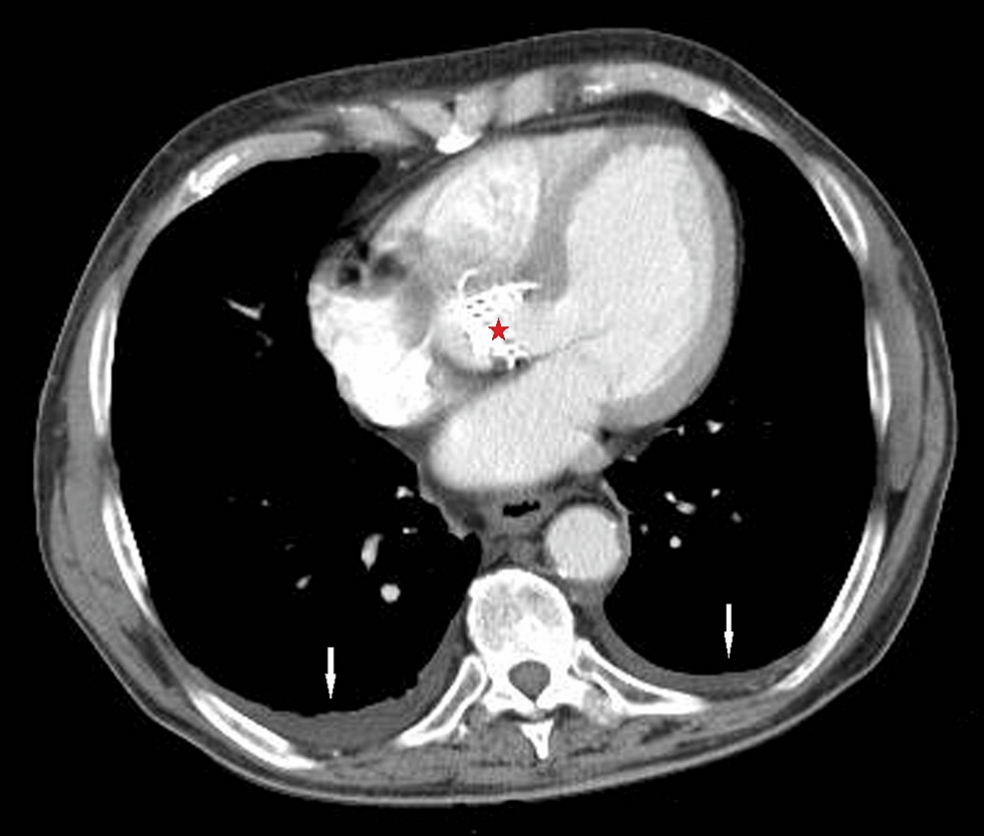
Chest CT scan, where the bioprosthetic aortic valve is visible (red asterisk), showing bilateral pleural effusions (white arrows).

**Figure 2:**
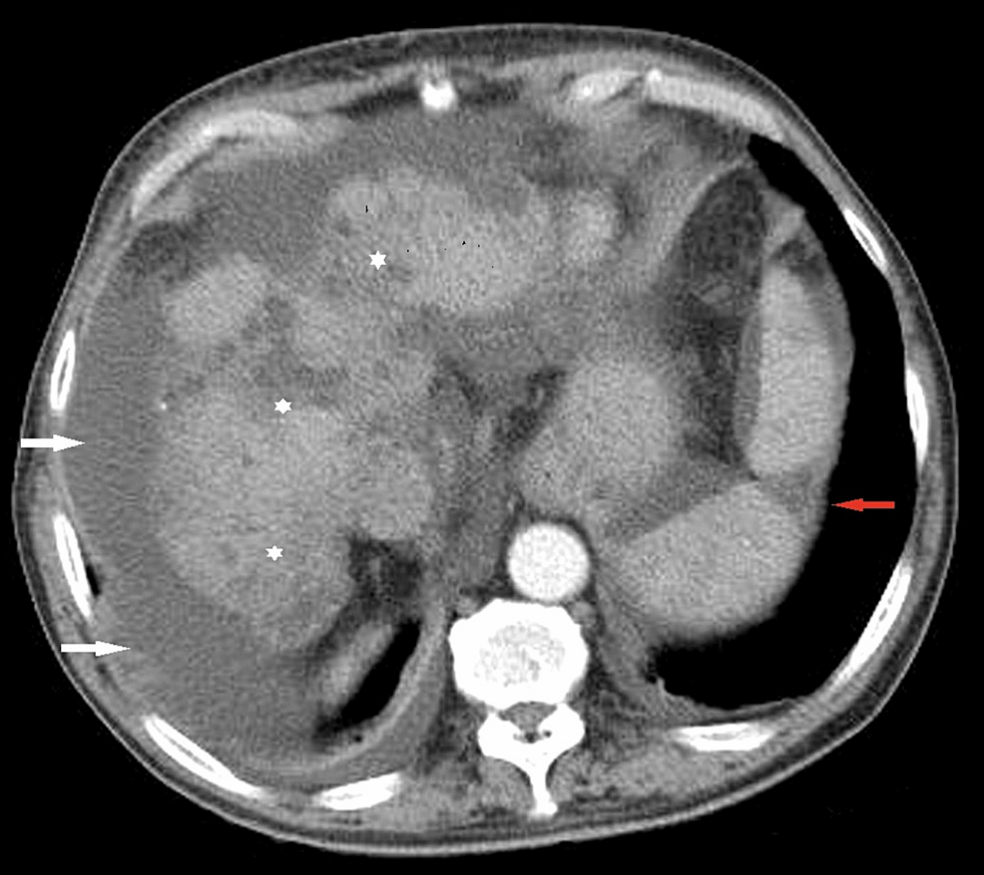
Abdominal CT scan showing a highly cirrhotic liver with surface nodularity and parenchymal heterogeneity (white asterisks) and ascitic fluid (white arrows). The triangular hypodense area indicates the splenic infarction (red arrow).

**Figure 3:**
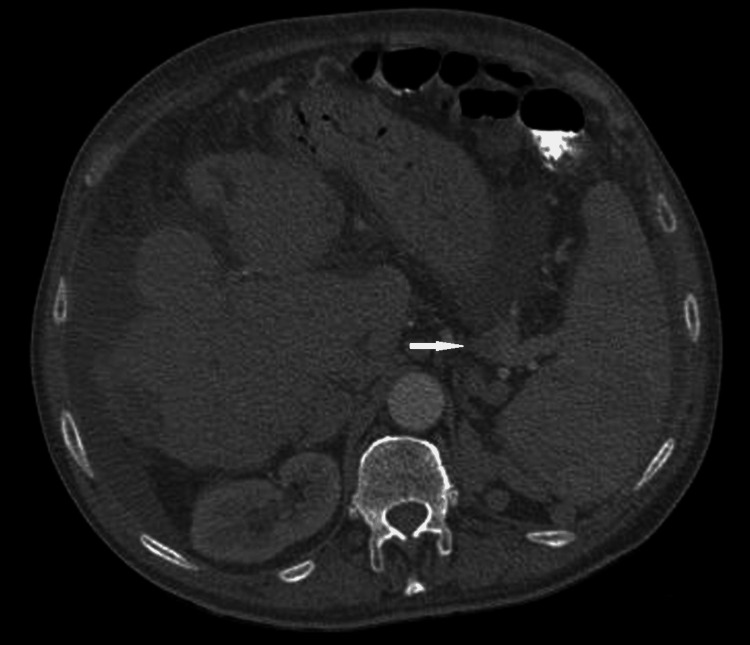
Abdominal CT scan showing splenic hilum varices (white arrow).

The transthoracic echocardiogram reported an ejection fraction of 50%, without wall motion abnormalities or pericardial effusion. No vegetation was detected, while the bioprosthetic aortic valve was fully functional and presented no leakage in the Doppler study. Urine culture yielded negative results. The chest CT scan revealed minor bilateral pleural effusions (Figure 1) and the abdominal CT scan confirmed the presence of ascites, a patent portal vein with varicose collaterals, and a hypodense area in the splenic parenchyma, attributed to a septic infarct (Figures 2, 3). No abscess formation or any sign of wall infection in the pre-existing aortic aneurysm was noted.

Taking into account the history of recent TAVI and the detection of no other obvious infection site in whole-body CT imaging, TAVI endocarditis was considered the most likely diagnosis, in accordance with meeting multiple modified Duke’s criteria. Our patient had sustained bacteremia, a predisposing cardiac lesion, high-grade fever (> 38^o^C), and splenic infarction detected on CT imaging. Due to the presence of esophageal varicose veins and the risk of bleeding, TEE was not performed [2,14,15]. Unfortunately, for administrative reasons, a PET-CT scan could not be arranged at that time.

Upon admission, empirical administration of teicoplanin had been initiated. No clinical improvement was noted with regard to the high fever for the following two days when blood culture results became available. *P. pentosaceus* was identified by the automated system (VITEK® 2 Compact, bioMérieux) and vancomycin resistance was confirmed by agar disk diffusion testing. Teicoplanin was discontinued and replaced by linezolid. Whereas fever and inflammatory markers improved shortly after the modification of antimicrobial therapy, a marked drop in the platelet count was noted after two weeks of hospitalization, attributed to linezolid. At that point, infectious disease consultation advised modification of the patient's regimen to piperacillin-tazobactam. We opted for adjunctive rifampicin treatment to reduce the risk of treatment failure, given the patient’s multiple co-existing conditions.

A new 5-fold increase in hsTnI values was observed, without chest pain or concomitant ECG changes. Cardiologic re-evaluation, again, reported no wall motion abnormalities or decrease in EF, with the exam being negative for the presence of vegetations. hsTnI elevation was attributed to the underlying infection and conservative treatment with close monitoring of cardiac enzymes was advised.

Our patient responded gradually, remaining afebrile, and zeroing inflammatory markers under the aforementioned regimen. hsTnI slowly normalized as well. Three more blood cultures were drawn, and all came back negative. After five total weeks of hospitalization, our patient was discharged with instructions to continue an oral combination of amoxicillin-clavulanic acid, rifampicin, and minocycline for an additional month. Laboratory results on the day of discharge are summarized in Table 2.

**Table 2:** Patient blood test results on the day of discharge. Pathologic results are presented in bold.

Lab parameter	Value	Reference ranges
Hemoglobin (Hgb)	10.7 g/dL	14-18 (for males)
Hematocrit (Hct)	32.8 %	42-52 (for males)
White blood cells (WBC)	3.00 K/μL	4,5-10
Neutrophils	1.02 (34.1%) K/μL	1.8-7
Lymphocytes	0.56 (22.1%) K/μL	1.2-3.8
Platelets (PLT)	81 K/μL	140-450
Aspartate aminotransferase (AST)	34 ΙU/L	0-40
Alanine aminotransferase (ALT)	20 IU/L	<45
Αlkaline phosphatase (ALP)	127 IU/L	30-120
γ-Glutamyl transferase (γ-GT)	36 IU/L	10-49
Total bilirubin	2.5 mg/dL	< 1.2
Direct bilirubin	0.7 mg/dl	<0.3 mg/dl
Indirect bilirubin	1.8	
Albumin (ALB)	4 gr/dL	3.4-4.8
Creatinine (Cre)	0.7 mg/dL	0.7-1.4
Urea	35 mg/dL	10-50
Sodium (Na^+^)	137 mmol/L	136-145
Potassium (K^+ ^)	4.1 mmol/L	3.5-5.2
High-sensitivity Troponin I (HsTnI)	30.3 pg/mL	<34.2 (for males)
C-reactive protein (CRP)	1.5 mg/dL	<0.7
Erythrocyte sedimentation rate (ESR)	7 mm/Hr	<30
International normalized ratio (INR)	1.1	0.8-1.1
Prothrombin time (PT)	11.9 sec	9.1-12.1

PET-CT can be used for evaluating treatment response in cases of endocarditis [3]. Upon completion of a total of eight weeks of treatment, 2-deoxy-2-[fluorine-18]fluoro-D-glucose positron emission tomography-computed tomography (18F-FDG-PET-CT) was scheduled to confirm the resolution of endocarditis. This was deemed necessary, given the patient’s comorbidities and the fact that the treatment plan was based on literature antimicrobial susceptibility data rather than an actual antibiogram. No pathological uptake was reported by the endocardium and the spleen, where septic embolism had been previously detected. On follow-up, the patient remains in good clinical condition, without symptom recurrence.

## Discussion

Microbiology and clinical significance

*Pediococci* are Gram-positive, α-hemolytic, or non-hemolytic, facultatively anaerobic cocci that produce pediocins and are normally found in fermented food [8,9]. They usually appear in pairs or tetrads upon microscopic inspection of blood agar [8]. They are a member of the LAB family (along with *Leuconostoc spp, Lactobacilli spp,* and *Enterococcus spp*.) and are often misidentified as S. viridans due to reaction with group D antisera [9,16-18]. 

Substantial biological benefits arise from the genus *Pediococcus*, such as the bactericidal effect of these cocci against common pathogens (i.e. *Listeria monocytogenes* and *Salmonella spp*.), effectuated through bacteriocin secretion [9]. *P. pentosaceus* strains have been increasingly utilized in the food industry as additives, providing flavor enhancement and facilitating the storage of meat, alcoholic beverages, and dairy products [7,19]. Since *P. pentosaceus* has been isolated from both the human oral cavity and stool, it is considered part of the microbiota colonizing the alimentary tract, with little or unknown pathogenic significance in the healthy host. *P. pentosaceus* can reduce oxidative stress in gut microbiota, demonstrating anti-inflammatory effects, and has been tested as a potential option to delay liver fibrosis in non-alcoholic fatty liver disease [20].

Among many species in the *Pediococcus* genus, *P. acidallactici* and P. pentosaceus have been the only ones increasingly isolated from specimens (including saliva, blood, stool, urine, and catheter tips) in both immunocompetent and, especially, immunocompromised patients with symptoms of infection [8,16]. Select cases have demonstrated its possible role as an opportunistic pathogen in immunocompromised hosts [19,21]. Long-term or frequent hospitalizations and exposure to vancomycin and other broad-spectrum antimicrobials play a role in the dysregulation of gut microbiota and the development of vancomycin-resistant LAB (especially *Enterococci*) [12,22,23].

Pediococcal septicemia was first reported in 1990 by Golledge et al. in a patient with leukemia [10]. Cases of bacteremia have been reported in a broad spectrum of ages, from infants and pregnant women to the elderly [11,18,24-27]. Clinical manifestations range from bacteremia and abscess formation to septic shock. Cases of peritonitis, urogenital and alimentary tract infections, and endocarditis have, also, been described [28-33].

Insertion of central venous catheters and gastrostomy tubes, neutropenia, and prior intra-abdominal interventions are listed among known predisposing factors for Pediococcus infection [8,25,34]. However, isolation of *Pediococci *from mixed cultures is common, making differentiation between cases of contamination and true pathogenicity challenging. In our case,* P. pentosaceus *was the only organism isolated from two sets of blood cultures drawn 10 days apart, before treatment modification led to its eradication in subsequent cultures.

Our patient’s main predisposing condition was liver cirrhosis, secondary to PBC, an autoimmune disorder mainly affecting middle-aged women [35]. Immune deficiency in cirrhosis is a complex and multifactorial phenomenon. Small intestinal bacterial overgrowth and intermittent bacterial translocation of dysregulated GI microbiota into the systemic circulation is well-established in cirrhotic patients and is attributed to pathologically increased permeability of the blood-gut barrier [36-38]. This explains the predominance of Gram-negative bacteria in infections common in cirrhosis, such as spontaneous bacterial peritonitis and blood-stream infections. Other recognized mechanisms of immune dysfunction in cirrhotic patients pertain to reduced complement factor synthesis, defective opsonization, and impaired function combined with increased apoptosis of all immune cells, including reticuloendothelial system cells [37]. The combination of gut dysbiosis, attenuation of gut barrier function, and immune cell dysfunction results in episodes of intermittent bacteremia [13,37]. Simultaneously, increased apoptosis and reduced proliferation of T-cells result in ineffective pathogen killing [13]. 

In 2012, Papanikolaou et al. reported *P. pentosaceus* isolation in blood cultures of an ICU patient, who became septic after administration of a total parenteral nutrition formula containing LAB [33]. Few other cases of variable infections triggered by probiotic administration, including a case of IE in a cirrhotic patient, have been reported [39,40]. In contrast to these cases, our patient had not received any probiotic supplements, either parenterally or per os. Furthermore, he did not report increased consumption of fermented meals. Probiotic administration in cirrhosis should be individualized but is generally considered a safe option [41]. Future randomized studies are required to further address this topic.

Antimicrobial susceptibility and therapeutic repercussions

*Pediococci* demonstrates inherent resistance to vancomycin, while teicoplanin exhibits lower minimal inhibitory concentrations (MIC) and better tissue penetration compared to vancomycin [8,22,23,42]. The mechanism of glycopeptide resistance in *Pediococci* is based on the termination of peptidoglycan wall precursors in a D-Ala-D-lactate sequence, while vancomycin binds to a D-alanine-D-alanine terminal [22]. Moreover, *Pediococci* acquires plasmid resistance genes against broad-spectrum antimicrobials.

Penicillin agents have shown good efficiency against *P. pentosaceus*, including anti-pseudomonal piperacillin [34,43-45]. In the clinical setting, clindamycin may be used as an alternative in patients allergic to penicillin [33,34]. Among other beta-lactam agents, imipenem has shown universally excellent efficiency, with MIC_90_ as low as 0.12 mg/L [34]. High rates of susceptibility to daptomycin have been exhibited in vitro, with MIC_90_ reported <0.5 mg/L [34,42,45,46]. Indeed, daptomycin carries bactericidal properties against vancomycin-resistant, Gram-positive bacteria and has been used successfully in cases of endocarditis and bacteremia caused by *P. acidallactici* and *P. pentosaceus*, respectively [21,25]. Finally, rifampicin and linezolid have both demonstrated good efficacy against* Pediococci *[34,43].

In our case, the hospital laboratory could only inform us about vancomycin resistance of the *Pediococcus* strain isolated from the patient’s blood. Reliable antimicrobial susceptibility testing may not be readily available for unusual and opportunistic pathogens in clinical practice, leading to difficulties in treatment decision-making in complex clinical situations. In such instances, literature microbial sensitivity data could serve as a guide in treatment planning.

The decision to add rifampicin to treatment was based on literature data on *Pediococcus* antimicrobial sensitivity, as analyzed above. Rifampicin has excellent penetrative properties, achieving high intracellular concentration, is well absorbed orally, and has been extensively used in prosthetic valve infections, especially when *Staphylococci* are involved [47-49]. Rifampicin is not used in isolation, due to the risk of rapid resistance development, but may be a useful adjective antimicrobial agent in gram-positive biofilm-forming infections, due to its action on non-dividing microbial cells found in biofilms [49-52]. IE in cirrhotic patients most commonly affects the aortic valve [53]. Given that this condition is characterized by a high risk of treatment failure and ensuing mortality, we decided to add rifampicin to the regimen, for its synergistic action against Gram-positive microbial biofilm, with careful monitoring of the patient’s liver function [53,54].

Due to the potential hepatotoxic effects of rifampicin, caution is warranted when the drug is administered to cirrhotic patients. There is extensive clinical experience derived from cirrhotic patients treated for tuberculosis, for which rifampicin is a critical drug [55,56]. In this case, weekly liver function tests for the first eight weeks of treatment, followed by monthly testing, is a reasonable approach and the one chosen by our patient. No hepatotoxicity was noted during his hospitalization. We also carried out appropriate adjusting of the patient’s other medication to avoid drug interactions with rifampicin, which is a cytochrome P-450 inducer.

The susceptibility of *Pediococcus spp*. to other antimicrobial classes has been studied extensively [34,42,44,46]. Results may vary slightly among studies, due to differences in methodology and the media used. Cephalosporins are not recommended as first-line treatment, due to disappointing in vitro results for first-, second- and third-generation cephalosporins [34,42]. Although aminoglycosides act synergistically with beta-lactamic antimicrobials, shortening the duration of treatment due to a stronger bactericidal effect, overall results of this class against *Pedioccocci *have been poor [8,43]. Within the class of aminoglycosides, gentamicin has been shown to be the most effective. Tetracycline susceptibility is limited, with the exception of minocycline [34,46]. Erythromycin has been shown to be effective in vitro, however, there is little data regarding its use in the clinical setting [34,42]. Susceptibility to other commonly used macrolides is not established.

Fluoroquinolone susceptibility is limited, with ciprofloxacin MIC_90_ measured as high as 32 mg/L in some studies [33,34]. In our case, the patient had initially responded to ciprofloxacin treatment as an outpatient, but fever rebounded after the regimen conclusion. A case of P. pentosaceus bacteremia in a patient with end-stage renal disease on dialysis, which was treated with ciprofloxacin has been reported [30].

Duke’s criteria in TAVI endocarditis

Dukes’ criteria fail to recognize up to 24% of cases of PVE. In TAVI endocarditis, echocardiography often fails to demonstrate valvular vegetation. 18F-FDG-PET-CT has the ability to detect infections on prosthetic valves, as well as other implanted heart devices and vascular grafts. According to the recent ESC guidelines, 18F-FDG-PET-CT has a class IB level of recommendation in the confirmation of PVE [3]. Its sensitivity and specificity for PVE have been shown to exceed 84% in a recent meta-analysis, partly due to its ability to neutralize artifacts affecting echocardiography [57,58]. However, despite being a useful diagnostic tool, PET-CΤ is subject to availability and cost restrictions.

## Conclusions

To our knowledge, this is the first reported case of *P. pentosaceus* TAVI endocarditis in a cirrhotic patient. Post-TAVI endocarditis poses a major clinical challenge, given the low sensitivity of Duke's criteria in TAVI patients and the requirement of markedly prolonged treatment. Additionally, TAVI patients commonly have significant underlying comorbidities and/or attenuated immune responses. These render them susceptible to bacteremia caused by unusual pathogens, which may affect the valve. In the absence of an antibiogram, antimicrobial susceptibility data documented in the literature may be used to successfully guide treatment. A multidisciplinary approach and use of advanced imaging techniques may contribute to guiding clinical decision-making and achieving optimal therapeutic results.
